# Past pandemics and climate variability across the Mediterranean

**DOI:** 10.1007/s41207-020-00197-5

**Published:** 2020-09-19

**Authors:** J. Luterbacher, T. P. Newfield, E. Xoplaki, E. Nowatzki, N. Luther, M. Zhang, N. Khelifi

**Affiliations:** 1 Science and Innovation Department, World Meteorological Organization (WMO), 7bis Avenue de la Paix, 1211 Geneva, Switzerland; 2 Department of History, Georgetown University, 37th and O Streets NW, ICC, Washington, DC USA; 3 Department of Biology, Georgetown University, 37th and O Streets NW, ICC, Washington, DC USA; 4 Department of Geography, Climatology, Climate Dynamics and Climate Change, Justus Liebig University of Giessen, Senckenbergstrasse 1, 35390 Giessen, Germany; 5 Centre of International Development and Environmental Research, Justus Liebig University of Giessen, Senckenbergstrasse 3, 35390 Giessen, Germany; 6 Earth and Environmental Sciences Editorial Department, Springer, a Part of Springer Nature, Tiergartenstrasse 17, 69121 Heidelberg, Germany

## Abstract

The influence that meteorological, climatological and environmental factors had on historical disease outbreaks is often speculated upon, but little investigated. Here, we explore potential associations between pandemic disease and climate over the last 2,500 years in Mediterranean history, focusing on ancient disease outbreaks and the Justinianic plague in particular. We underscore variation in the quality, quantity and interpretation of written evidence and proxy information from natural archives, the comlexity of identifying and disentangling past climatological and environmental drivers, and the need to integrate diverse methodologies to discern past climate-disease linkages and leverage historical experiences to prepare for the rapid expansion of novel pathogenic diseases. Although the difficulties entailed in establishing historical climate-pandemic linkages persist to the present, this is a research area as urgent as it is complex and historical perspectives are desperately needed.

## Plagues and climate in Mediterranean history, some initial steps

The dynamics of the ongoing COVID-19 pandemic are an urgent research area of great importance to governments across the Mediterranean. Decisions of significant public health and economic consequence are influenced by epidemiological forecasts and spread-modifying factors associated with the physical environment (COVID-19 e-symposium outcome statement, 2020). Meteorological, climatological and environmental factors may influence SARS-CoV-2 transmission and may have contributed to its emergence, though current evidence is not yet consistent or conclusive (COVID-19 e-symposium outcome statement, 2020). Although the interdisciplinary research required to untangle complex relationships between climate and disease outbreaks could benefit from historical perspectives, available data on past climate-disease linkages are of shallow temporal depth and many evidential gaps remain to be overcome. Here we begin to explore pandemic disease’s associations with climate and environmental changes in Mediterranean history. We underscore variation in the quality, quantity and interpretation of written evidence, the complexity of identifying and disentangling past climatological and environmental drivers, and the need to integrate diverse methodologies if we are to leverage historical experiences to prepare for the rapid expansion of novel pathogenic diseases.

Roughly 2450 years before SARS-CoV-2 made its appearance in the Mediterranean region, Thucydides (1956 II.47–54) authored an account of a plague that had commenced, he claimed, in East Africa before spreading in the eastern Mediterranean and devastating his home *polis*. Cryptic references to Sumerian, Hittite and Biblical disease outbreaks aside, many historians consider this Athenian Plague, 430–27 BCE, among the first interregional disease outbreaks, pandemics, to affect the Mediterranean. The Antonine, 165–89 CE, Cyprianic, 250–70 CE, and Justinianic, 541–44 CE, plagues (Morgan 1994; Gilliam 1961, 1996; Greenberg 2003; Harper 2015; Sarris 2002; Mordechai et al. 2019) followed. Eight centuries then passed before the Black Death, 1346–53 CE, the greatest pandemic in recorded world history, quickly killed tens of millions of people across the Mediterranean and far beyond (Biraben 1975; Benedictow 2004; Green 2014). Like the Justinianic plague, the Black Death marked the arrival in the region of *Yersinia pestis*, the cause of bubonic, pneumonic and other varieties of plague. For more than two centuries following the 6th-century pandemic, and five following the 14th-century pandemic, *Y. pestis* would repeatedly reemerge within the Mediterranean region and Europe, often claiming vast numbers of lives, as in Egypt 1429–30 CE, Spain 1596–1602 CE and Italy 1629–31 CE (Biraben 1975; Stathakopoulos 2004; Borsch 2005; Alfani 2017; MacKay 2019; Varlik 2014).

That our current understanding of these outbreaks is, like the foregoing sketch of Mediterranean disease outbreaks, enormously incomplete, considerably complicates attempts to establish connections between them and climate. The chronology and geography of the earliest plagues are poorly understood, so too their pathogenic identity. Most diagnoses of prelaboratory plagues are debated, with the exception of those of the Justinianic plague and Black Death, for which we now have confident DNA-based *Y. pestis* identifications (Bos et al. 2011; Spyrou et al. 2019; Keller et al. 2019a, b). That the earliest of the aforementioned plagues were the most demographically or socioeconomically significant ones to afflict the ancient Mediterranean is itself uncertain. Some doubt whether the Antonine plague was a pandemic at all and the construction of the Cyprianic plague begs for closer scrutiny. For all we know, other outbreaks, more poorly documented yet, like the “deadly epidemic” of 65 CE, which purportedly killed 30,000 in Rome in a single autumn alone and left “houses filled with lifeless bodies and streets with funerals” (Tacitus 1962 XVI.13; Suetonius Nero 1914 XXXIX.1), could have been more disruptive. Between the last known Justinianic recurrence and the Black Death (~ 750–1345 CE), other disease outbreaks, possibly significant but hitherto ignored, occurred too (Schnerrer 1823; Curschmann 1900). There is much to do in Mediterranean disease history.

Whether the aforementioned plagues represent novel disease emergences or the introduction of a disease endemic elsewhere is important. Unsurprisingly, our understanding of historical pandemic origins remains poor. The Antonine plague may have emerged in North Africa or Southwest Asia, and the Cyprianic plague in East Africa or the Pontic Steppe; alternatively, these regions may simply be where these plagues entered the purview of literate observers. The alleged East African origins of many ancient plagues (Gilliam 1961, 1996; Harper 2015; Allen 1979) may owe little more than to Greco-Roman prejudices and Thucydides’ influence, but few Mediterranean plagues may have been limited to the Mediterranean region itself; some, like the Justinianic plague and Black Death, were more global in scope.

**Fig. 1:**
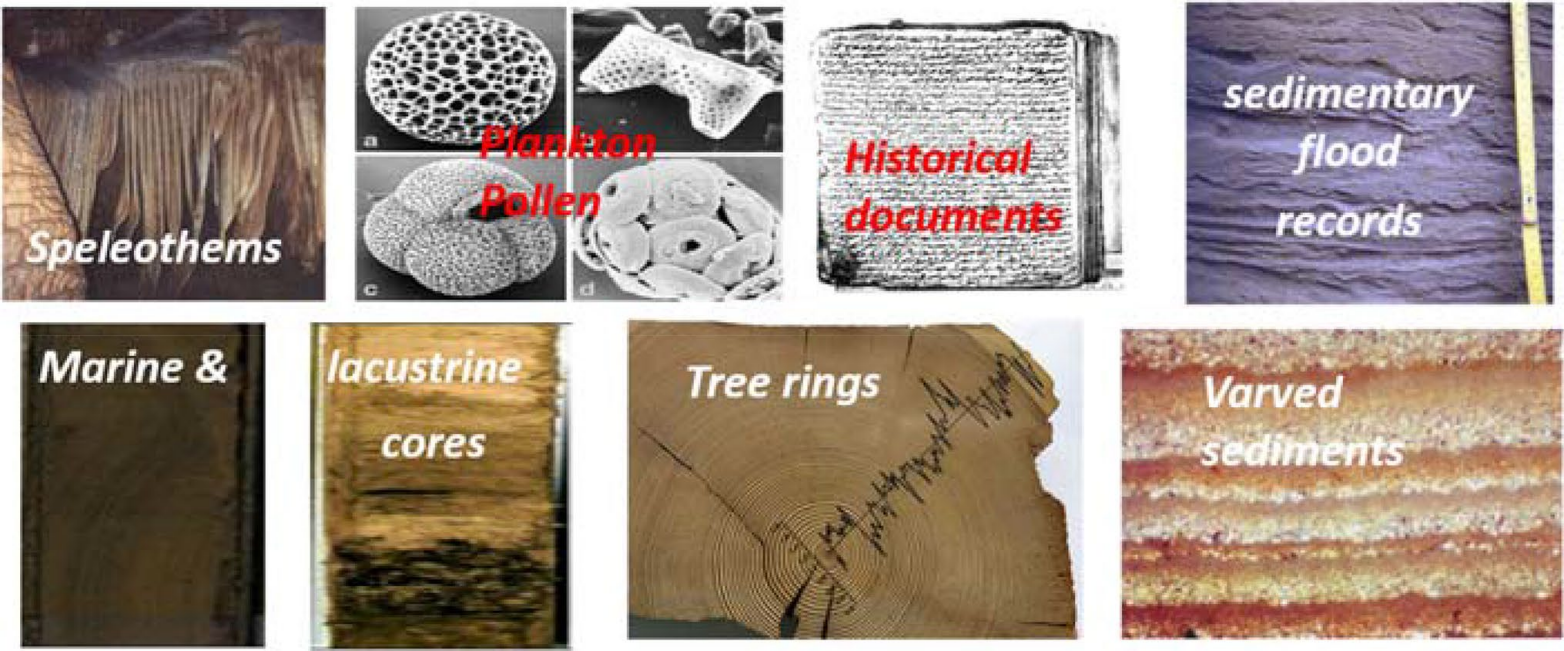
Examples of documentary and natural proxies for palaeoclimate and environmental research in the Mediterranean (adapted from Bradley 2015)

A number of scholars have attempted to untangle past climate–plague relationships (Stothers 1999; Rossignol and Durost 2007; Schmid et al. 2015; Elliott 2016; Campbell 2017; Green 2018; Newfield 2018a). To establish whether climatological and environmental factors influenced the emergence of past Mediterranean pandemics knowing where and when the outbreak began, and its pathogenic identity, are fundamental, as are high quality, long-running climate records from different archives, which resolve various aspects of climate and environment change at high spatial and temporal resolution and which also cover the full annual cycle. Although the Mediterranean region offers an unusually rich combination of natural archives (terrestrial and marine proxies, including tree-rings, speleothems, lake, river and marine sediments, and vermetids), as well as comparatively plentiful, albeit spatiotemporally variable and highly heterogeneous, documentary evidence (Fig. 1; Bradley 2015; Luterbacher et al. 2012; Finné et al. 2019; Labuhn et al. 2018; Xoplaki et al. 2018), records extending back to antiquity remain limited.

**Fig. 2:**
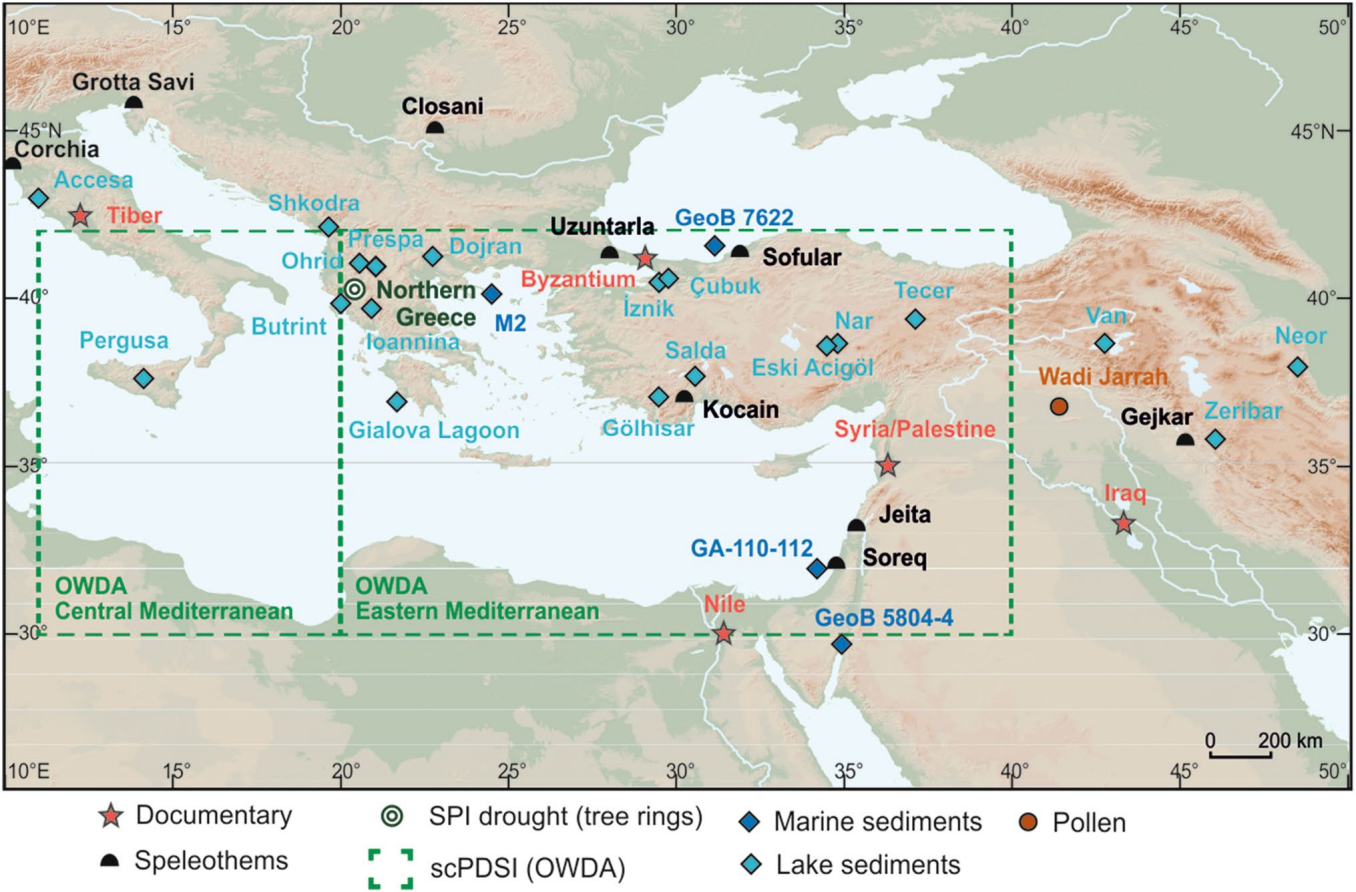
Central and eastern Mediterranean records for hydroclimate/temperature from natural and select documentary sources (central Italy, Byzantium, Iraq and the lower Nile), shown by proxy type and covering all, or a large part of, the past 1-to-2 millennia (from Luterbacher et al. 2021, in press)

Not only is knowledge of Mediterranean climate during the aforementioned plagues remarkably incomplete, but also high-resolution climate proxies, which are required to discern potential climate-disease linkages, are particularly scarce. Coverage for different seasons and variables (temperature, precipitation, drought, sea level changes, pH, sea water temperature, water mass circulation, etc.) are also uneven. Figure 2 plots the range of climate evidence from various proxy records available for the central and eastern Mediterranean region and covering the past one-to-two millennia (Luterbacher et al. 2021, in press).

As tricky as establishing climate-pandemic correlations may be, teasing out causal linkages is far more difficult. Climate and disease interact in complex ways and simultaneously through cultural and environmental systems as well as social and economic structures. The effects of climate change on pandemics will vary accordingly between and within regions and evolve as an outbreak persists, from emergence to cessation. Concern for temporal and spatial scales, of climate and disease, is consequently vital and broad-stroke sketches of historical linkages (Keys 1999; McMichael 2011; Harper 2017), particularly for past pandemics for which evidence is still partial, are certain to mislead. While causal mechanics are instead to be discerned in more small-scale analyses (Stenseth et al. 2006; Ben Ari et al. 2011; Haldon 2016), that climate and disease will have interacted historically at various points, and on various temporal and spatial scales, within one outbreak remains a major research challenge.

Climate may shape the course of a disease outbreak indirectly via its influence on vegetation and agroecosystems, and, for instance, on the cascading effects stemming from failed harvests, such as migration for food and work, malnutrition and compromised immune function. It can concurrently intersect with disease ecology and transmission, affecting host and vector populations and human behaviour in ways that alter pathogen dissemination and disease severity. Usually cool conditions might cause more people to spend more time indoors, which in crowded habilitations could facilitate the dissemination of respiratory pathogens and diseases hosted by commensal rodents or arthropods (Neher et al. 2020). Without an understanding of these factors, causal climate-disease mechanisms will remain challenging to establish. We must also stay cognizant of the influence of climate variability on endemic infectious disease in regions in which an outbreak spreads, as alterations to preexisting disease landscapes would affect outbreak outcomes. The same rainfall-temperature variation that facilitates a plague spillover in a given region (Stenseth et al. 2006; Ben Ari et al. 2011) might also, for instance, meaningfully diminish populations of anopheles mosquitoes which spread malaria and consequently limit malaria-plague coinfections (Newfield 2017).

**Fig. 3:**
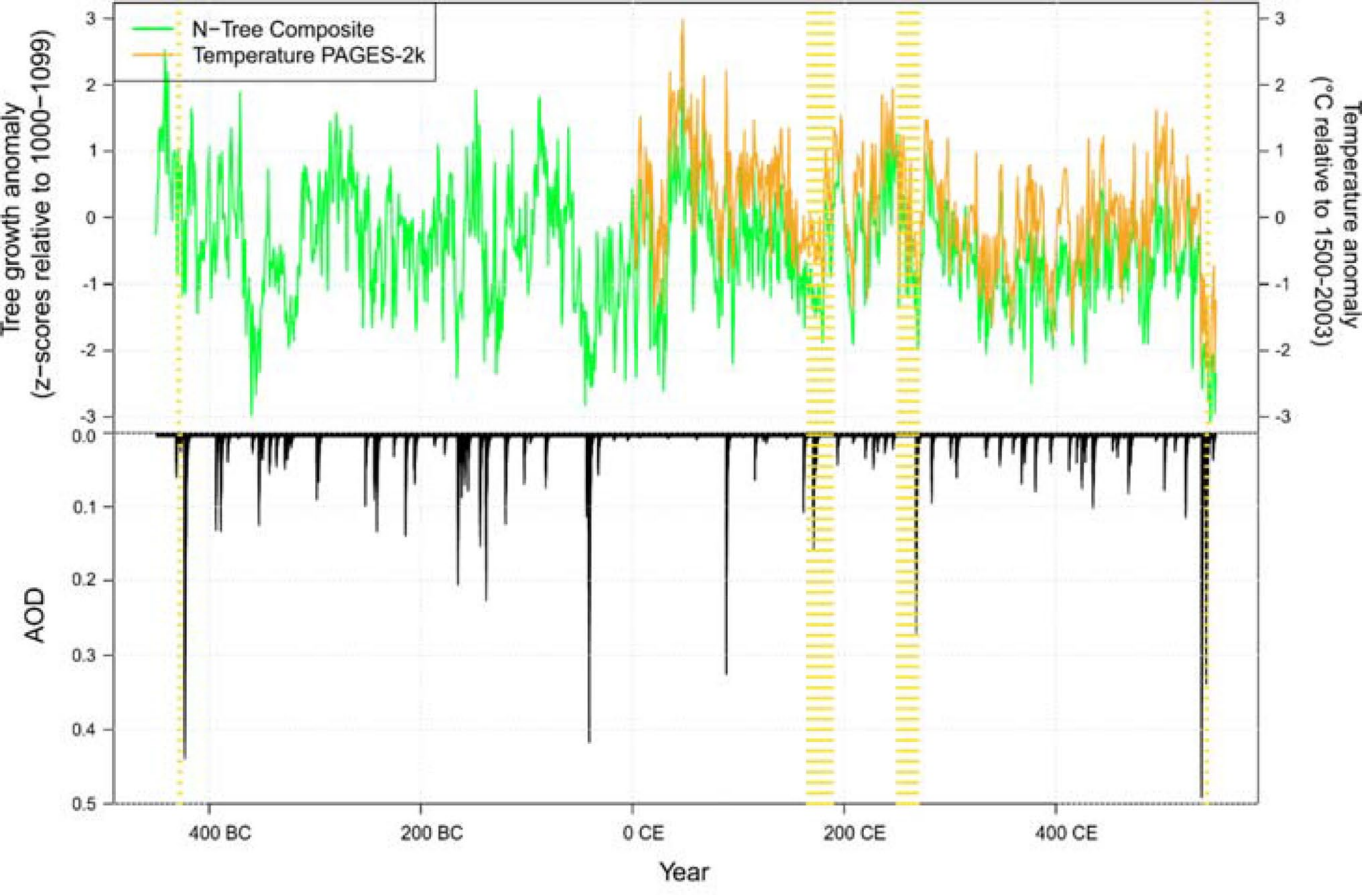
N-Tree Composite: a multi-millennial, summer temperature sensitive composite of tree-ring records spanning the Northern Hemisphere with a strong bias to JJA temperatures (anomalies w.r.t. 1000–1099 CE; from Sigl et al. 2015, their Fig. 3), here displaying 450 BCE–550 CE; Temperature Pages-2k: European summer temperature anomalies, reconstructed from tree rings, for the first 5,5 centuries (PAGES-2k 2013; Luterbacher et al. 2016) relative to 1500–2003 CE; AOD: estimated global stratospheric Aerosol Optical Depth of large volcanic eruptions derived from polar ice-core sulfate records (Toohey and Sigl 2017); Yellow-hatched bars represent the duration, as commonly understood, of the Athenian, Antonine, Cyprian and Justinianic plagues

Untangling historical pandemic-climate linkages is plainly no easy task. Consider that the four earliest aforementioned plagues each temporally correspond to some of the largest climate-forcing volcanic eruptions of the last 2500 years (Fig. 3). Tempting as it may be to link these plagues to these eruptions, correlation and causality by no means necessarily go hand-and-hand.

While the Athenian, Antonine, Cyprianic and Justinianic plagues each correlate to an unusually large, climate-forcing eruption, we do not yet possess the data and understanding of these events we need to connect them. The unidentified eruptions of ~ 426 BCE, ~ 160 CE and ~ 169 CE, and ~ 266 CE, like the eruptions of 536 CE and 540 CE (Büntgen et al. 2016; Newfield 2018b), clearly impacted Northern Hemispheric tree-ring based reconstructed summer temperatures. However, whether such seasonally-based temperature variability facilitated the emergence or diffusion of these outbreaks is, without definitive diagnoses and a clear grasp of spatiotemporal parameters, unclear.

There was, evidently, no simple relationship between summer cooling and ancient Mediterranean pandemic disease, as several pronounced cooling periods do not correspond to known large-scale disease outbreaks, including the cooling associated with the massive eruption of 43 BCE (McConnell et al. 2020). Weary as we should be of drawing macro-level climate-pandemic linkages on the basis of current evidence, that significant summer cooling contributed to pandemic emergence beyond the Mediterranean region, whether in Africa, Asia or Europe, and facilitated Mediterranean disease diffusion, indirectly through a combination of mechanisms, remains a possibility, as plague-cooling concurrences visualised in Fig. 3 may suggest. Yet, for all we presently know, significant summer cooling, volcanically forced or not, may have actually served to limit the spread of rodent-borne or arthropod-vectored pandemics (Newfield 2018a, b). Major volcanic events and corresponding summer cooling do seem to occur at the tail end of both the Athenian and Cyprianic plagues. At the same time, the diagnoses and epidemiology of those plagues are to be determined and whether that cooling occurred on a local scale in plague-affected regions, like Athens, is entirely unclear without sufficiently long-running Mediterranean climate proxies that resolve intraannual variability.

Of ancient pandemics, we know most about the Justinianic plague, though uncertainties still complicate potential climate and environmental linkages. Remnants of *Y. pestis* have been identified in the remains of ~ 45 late antique individuals (Wiechmann and Grupe 2005; Harbeck et al. 2013; Wagner et al. 2014; Feldman et al. 2016; Keller et al. 2019a, b), confirming the symptom-based plague diagnosis of this pandemic. But the origins of the outbreak are murky. Most historians, following the 6th-century authority Procopius (1914 II.22–23), hold the pandemic enters recorded history in July 541 at Pelusium in the eastern Nile Delta, though other late antique sources suggest the disease appeared first in southern Southwest Asia or East Africa. The phylogeny of ten plague genomes reconstructed from late antique human plague victims (Harbeck et al. 2013; Wagner et al. 2014; Feldman et al. 2016; Keller et al. 2019a, b) indicate that the Justinianic plague *Y. pestis* lineage originated centuries earlier in Central Asia (possibly in the foothills of the Tian Shan mountains in modern-day Kyrgyzstan). In the emergence-pandemic interval, it has been hypothesized that *Y. pestis* focalised in East Africa (Green 2015; cf. Keys 1999) and irrupted as the Justinianic plague in that region not long before 541 CE and that it then either travelled north to the Red Sea port of Berenice, before transferring to the Nile and heading north, or to the port of Clysma, before entering the eastern Delta via the Amnis Traianus, an ancient canal connecting the Red Sea and eastern Nile Delta (Stathakopoulos 2004; McCormick 2007; Tsiamis et al. 2009). Whichever route, these theories take for certain Procopius’ identification of Pelusium as the Mediterranean city first hit and the Thucydidean claim of Evagrius (2000 IV.29), a late 6th-century historian, that the plague began in East Africa. John of Ephesus, who like Procopius lived through the Justinianic plague, discusses the pandemic first in Alexandria, but identifies populations south of Egypt and in Southern Arabia (former Himyarite and Kush lands) as struck early on (Michael the Syrian 1901 IX.28). Later texts write of the plague spreading through the eastern Mediterranean as well as East Africa and Persia, but do not specify which region was struck initially (Newfield 2018a).

As stressed, disease ecology and epidemiology are fundamental to establish climate-pandemic linkages. Plague is a complex disease (Dubyanskiy and Yeszhanov 2016; Jones et al. 2019). Over 200 species of rodents and 30 species of fleas are known to maintain *Y. pestis* today in tens of foci in more than 25 countries on four continents (Baril et al. 2019). Far from all current foci are centuries-old and many are doubtless extinct. The Justinianic plague may have emerged from a non-extant reservoir in East Africa or southern Arabia, where sixth-century writers identify the disease first, or farther afield yet, but small genetic variations in the ten late antique plague genomes presently available teach that recorded Justinianic plague recurrences in the Mediterranean region represent not *Y. pestis* reintroductions from afar, but re-emergences from one or more historical plague reservoirs in the Mediterranean region (Keller et al. 2019a; Tsiamis et al. 2011).

**Fig. 4:**
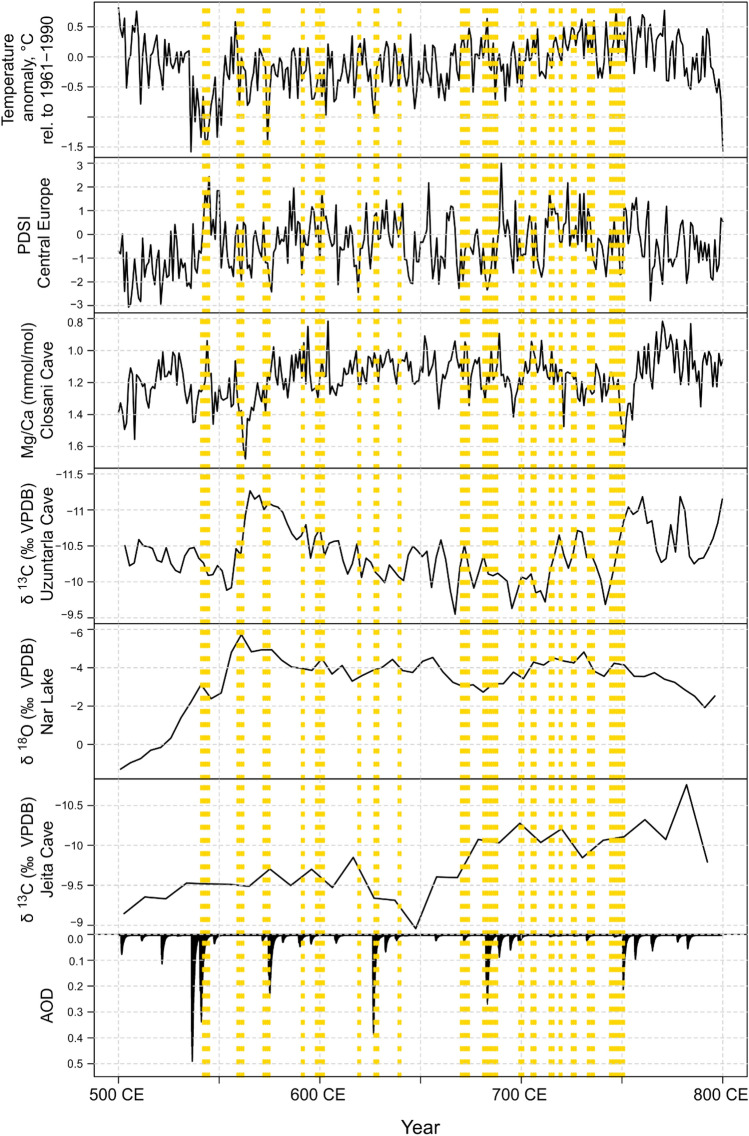
Tree-based reconstruction of European summer temperature anomalies (w.r.t. 1961-1990; Luterbacher et al. 2016); tree-based reconstruction of Central European summer precipitation using the Palmer Drought Severity Index (Cook et al. 2015); speleothem-based Mg/Ca record indicative of autumn winter precipitation from Closani, southwestern Romania (Warken et al. 2018); speleothem-based δ^13^O record partially indicative of hydroclimate from Uzuntarla, northwestern Turkey (Göktürk 2011); varved sediment-based record of δ^18^O indicative of winter-spring precipitation from Lake Nar, central Turkey (Jones et al. 2006); speleothem-based record of δ^13^C indicative of hydroclimate from Jeita, Lebanon (Cheng et al. 2015); estimated global stratospheric Aerosol Optical Depth (AOD) of large volcanic eruptions derived from polar ice-core sulfate records (Toohey and Sigl 2017). Yellow-hatched bars represent the Justinianic plague (541-544 CE) and record first plague pandemic recurrences. This plague chronology follows Stathakopoulos (2004), but the written record of late antique plague presents many challenges and other chronologies differ slightly (Tsiamis et al. 2011; Harper 2017)

Historians of the Black Death have speculated that marmots in the southern Alps, Jerboas in Anatolia and voles in England maintained second-pandemic plague (Carmichael 2014; Varlik 2014; Pribyl 2017), but what rodents might have hosted first-pandemic plague in the Mediterranean region remains uncertain. It has been emphasized that for plague to persist and cause human spillovers for centuries a plurality of rodents and fleas, sylvatic and commensal (Dubyanskiy and Yeszhanov 2016; Jones et al. 2019), would have been involved in any given region. That small ruminants and camels can maintain the disease as well could be another important factor (Malek et al. 2016; Dai et al. 2018). While repeated reemergences from regional rodent reservoirs may have been associated with climate, not knowing where plague focalised or what species were involved complicates attempts to discern a consistent ‘signal’, such as an unusually wet-humid growing season on the heels of a drought (Ben Ari et al. 2011), in available paleoclimate proxies. That the written record of late antique plagues is incomplete, the full spatiotemporal scope of every first-pandemic plague outbreak is unknown, and the chronology and scope of identifiable individual outbreaks is debated, are major additional confounding factors. Further, not every reemergence needs to have been climate triggered, nor need every climate-trigger spillover have spread widely. As visualized in Fig. 4, any simple correlation between plague reemergence and European summer temperatures (Luterbacher et al. 2016) or continental European summer precipitation is unapparent (Cook et al. 2015). Several selected regional proxies (see Fig. 2 for locations) similarly do not align tidily with the written record of first-pandemic plague recurrences, such as speleothem-based reconstructions of autumn-winter precipitation from Closani, southwestern Romania (Warken et al. 2018), of hydroclimate from Uzuntarla, northwestern Turkey (Göktürk 2011), or of the hydroclimate from Jeita, cental Lebanon (Cheng et al. 2015). Likewise, a sediment-based record of winter-spring precipitation from Lake Nar, central Turkey, does not correlate with known plague recurrences (Jones et al. 2006). The Uzuntarla and Nar records, which are subdecadally resolved but not annually, and the Jeita record, which is of lower resolution yet, makes obvious the importance of high spatio-temporal resolved proxies that also cover seasonal climate aspects. Identifying plague-climate linkages using a climate record dated at five-year intervals is understandably challenging. The largest late antique volcanic eruptions, and their associated forcing on Northern Hemispheric climate, each correspond to a plague outbreak, but the majority of outbreaks are associated neither with large eruptions nor summer cooling in Europe. Whether a pronounced multi-year megadrought archived in some speleothem and lake records from southern Arabia and northern Ethiopia 10-to-20 years before the appearance of the Justinianic plague in late antique texts (Fleitmann et al. 2007; Marshall et al. 2009) is relevant to the onset of the first plague pandemic is unclear, but is worth exploring further considering aforementioned arguments for a proximate origin of the Justinianic plague in either region.

A more concerted effort has been made recently to understand the Black Death in the context of climate change (Schmid et al. 2015; Campbell 2016; Green 2018). The focus has set again on the pandemic’s origins, which phylogenetic analyses of *Y. pestis* isolated from late medieval and early modern plague victims have come to inform. The pandemic reached the Mediterranean in late summer 1347 (Byzantine lands in August–September, Sicily in October, and Egypt and southern France in November) from the Black Sea and likely through Southwest Asia, having spread in the Il-Khanate in the 1340s and reached Baghdad in summer 1347 (Benedictow 2004; Barker 2021, Forthcoming). The outbreaks’s earlier geography is tough to establish, but it has been located next to Lake Issyk Kul in Kyrgyzstan in 1338 and in far-removed Azerbaijan, Crimea and Uzbekistan in 1346 (Benedictow 2004; Green 2018; Slavin 2019; Barker 2021). The date of the Black Death’s initial spillover is widely speculated. The emergence of the pandemic’s *Y. pestis *lineage is dated presently to ~ 1250 CE (Cui et al. 2013; Spyrou et al. 2019), and while some argue the pandemic commenced only ~ 10 years before it reached the purview of western Eurasian observers, others have proposed, recently and long ago, that plague spread earlier in East and Central Asia, as early as the mid-thirteenth century (Hecker 1832; Hymes 2014; Green 2014). Plague genomes also indicate that plague focalized in or near the Mediterranean region and Europe following the Black Death and reemerged regionally for centuries (Bos et al. 2016; Seifert et al. 2016; Spyrou et al. 2016, 2019; Namouchi et al. 2018), doing away with the epidemiological orientalism that informed earlier claims of repeated Mediterranean reintroductions from afar.

Yet, whether climate influenced initial emergence or subsequent Mediterranean-area spillovers remains an open question, as where and when precisely second-pandemic plague emerged, and the locations and species composition of any Mediterranean or European reservoirs, are uncertain. Connections hitherto drawn between the Black Death and the large, climate-forcing eruption of Samalas, Indonesia (1257; Guillet et al. 2017) or the early fourteenth-century European pluvial (Cook et al. 2015), are not robust. Finer dating and mapping of the pandemic’s emergence and closer attention to associated sylvatic rodent ecology in connection to perceptible climate change are much needed.

Regarding Mediterranean-region re-emergences, different foci with particular environmental and climate ties could have sourced outbreaks in any one region. Some reservoirs may not have lasted long. How meteorological, climatological and environmental variables shaped outbreaks post emergence is likewise an intricate issue, as mechanisms of second- (and first-) pandemic plague transmission could have varied spatially and temporally. Outbreaks could, for instance, involve commensal, semi-commensal and slyvatic rodents and their fleas, as well as, or perhaps only, human fleas and lice. Plague could as well spread pneumonically and gastrointestinally. Clearly, the obstacles we have yet to overcome with regard to climate’s possible influence on the Justinianic plague and the first plague pandemic likewise complicate our understanding of potential Black Death and second plague pandemic associations with climate.

## Persistent data challenges and ways forward

We are confronted with similar challenges today. The original and intermediate host species of SARS-CoV-2 are uncertain (the horseshoe bat and a pangolin species are, respectively, probable), as are where and when the intermediate host acquired the virus and SARS-CoV-2 jumped to humans (Anderson et al. 2020; Liu et al. 2020; Xiao et al. 2020). Sequence data from early samples suggests the novel coronavirus spilled over into people in mid-to-late November 2019 (Anderson et al. 2020; Dorp et al. 2020; Li et al. 2020), possibly weeks before the first identified case (Huang et al. 2020), but earlier unconfirmed cases confound the issue. With such a fragmentary grasp of key events, whether changing weather patterns facilitated the emergence of SARS-CoV-2 is imperceptible.

The difficulties entailed in establishing historical climate-pandemic linkages, of course, are far greater. Nevertheless, this is a research area as urgent as it is complex. Historical perspectives are desperately needed. Poor data availability and spatiotemporal resolution represent major challenges we must overcome. Gathering existing data and producing what are still needed to identify causal mechanics connecting weather, climate and environmental conditions to disease will take time, but has the potential to improve significantly not only our grasp of historical spillovers but also how meteorological, climatological and environmental changes contributes to the dissemination of known infectious diseases and the development of new ones. Interdisciplinary collaborations will be pivotal moving forward, so too case studies focused on periods, places and disease outbreaks for which high-resolution data and definitive or near-definitive diagnoses are obtainable. Importantly, case studies of major historical disease outbreaks, like those addressed here, may prove informative, but concentrating on recent historical periods may yield, for the reasons we have stressed, fuller climate-disease histories. Still, working across disciplines we can begin to reliably reconstruct climate’s influence on past disease and leverage that knowledge for epidemic preparedness and spillover risk assessments today.
